# Dissecting the predictive value of MAPK/AKT/estrogen-receptor phosphorylation axis in primary breast cancer to treatment response for tamoxifen over exemestane: a Translational Report of the Intergroup Exemestane Study (IES)—PathIES

**DOI:** 10.1007/s10549-018-05110-x

**Published:** 2019-01-24

**Authors:** Zsolt Szijgyarto, Koen D. Flach, Mark Opdam, Carlo Palmieri, Sabine C. Linn, Jelle Wesseling, Simak Ali, Judith M. Bliss, Maggie Chon U. Cheang, Wilbert Zwart, R. Charles Coombes

**Affiliations:** 1 Clinical Trials and Statistics Unit (ICR-CTSU), Division of Clinical Studies, The Institute of Cancer Research, London, SM2 5NG UK; 2 Division of Oncogenomics, Oncode Institute, The Netherlands Cancer Institute, 1066CX Amsterdam, The Netherlands; 3 Division of Molecular Pathology, The Netherlands Cancer Institute, Plesmanlaan 121, 1066CX Amsterdam, The Netherlands; 4 Department of Molecular and Clinical Cancer Medicine, University of Liverpool, Liverpool, L69 3BX UK; 5 Academic Department of Medical Oncology, Clatterbridge Cancer Centre NHS Foundation Trust, Wirral, CH63 4JY UK; 6 Department of Cancer and Surgery, Faculty of Medicine, Imperial College London, Du Cane Road, London, W12 0NN UK; 7 Department of Medical Onology, The Netherlands Cancer Institute, Plesmanlaan 121, 1066CX Amsterdam, The Netherlands; 8 Department of Pathology, University Medical Center Utrecht, Utrecht, The Netherlands; 9 Department of Pathology, The Netherlands Cancer Institute, Plesmanlaan 121, 1066CX Amsterdam, The Netherlands

**Keywords:** Breast cancer, Aromatase, Tamoxifen, Prognosis, Biomarkers

## Abstract

**Purpose:**

The prognostic and predictive values of the MAPK/AKT/ERα phosphorylation axis (pT202/T204MAPK, pT308AKT, pS473AKT, pS118ERα and pS167ERα) in primary tumours were assessed to determine whether these markers can differentiate between patient responses for switching adjuvant endocrine therapy after 2–3 years from tamoxifen to exemestane and continued tamoxifen monotherapy in the Intergroup Exemestane Study (IES).

**Methods:**

Of the 4724 patients in IES, 1506 were managed in a subset of centres (*N* = 89) participating in PathIES. These centres recruited 1282 (85%, 1282/1506) women into PathIES of whom 1036 had phospho-marker data. All phospho-markers were analysed by immunohistochemistry staining. Multivariable Cox proportional hazards models of the phospho-markers for disease-free survival (DFS) and overall survival (OS) were adjusted for clinicopathological factors. Treatment effects on the biomarker expression were determined by interaction tests. Benjamini–Hochberg adjustment for multiple testing with a false discovery rate of 10% was applied (*p*_BH_).

**Results:**

Phospho-T202/T204MAPK, pS118ERα and pS167ERα were all found to be correlated (*p*_BH_ = 0.0002). These markers were not associated with either DFS or OS when controlling for the established clinicopathological factors. Interaction terms between the phospho-markers and treatment strategies for either DFS or OS were not statistically significant (*p*_BH_ > 0.05 for all).

**Conclusions:**

This PathIES study confirmed previously described associations between the phosphorylation site markers of AKT, MAPK and ERα activity in postmenopausal breast cancer patients. No prognostic correlations between the phosphorylation markers and clinical outcome were found, nor were they predictive for clinical outcomes among patients who switched therapy over those treated with tamoxifen alone.

**Electronic supplementary material:**

The online version of this article (10.1007/s10549-018-05110-x) contains supplementary material, which is available to authorized users.

## Background

Globally, around 1.7 million new breast cancer cases are diagnosed each year, with over 550,000 patients who succumb to the disease [1]. The majority of cases (70–80%) are diagnosed with estrogen-receptor alpha (ERα)-positive disease and these patients routinely receive endocrine therapeutics as adjuvant treatment following surgery. The most commonly prescribed endocrine therapies in the adjuvant treatment of breast cancer are tamoxifen, or in postmenopausal women aromatase inhibitors (AIs) or sequential treatment of the two. The Intergroup Exemestane Study (IES) reported superiority of tamoxifen for 2–3 years followed by AIs, as compared to tamoxifen alone [2]. These findings were confirmed in a recent meta-analysis, which has shown that aromatase inhibitors, given at some point during the treatment (either at the start or after 2–3 years prior tamoxifen exposure) outperforms tamoxifen monotherapy [3].

Currently, it remains elusive whether suitable biomarkers can be identified that would facilitate optimal endocrine treatment selection in the adjuvant treatment of breast cancer, identifying individual patients who would derive selective benefit from tamoxifen, AIs or sequential treatment. Our previous analyses showed that high expression of ERß is indicative of no benefit in switching [4]. In contrast, high levels of cell proliferation marker Ki67 indicated selective benefit of AIs over tamoxifen alone [5].

Phosphorylation of ERα at serine residues 118 and 167 by MAPK and AKT, respectively, increases its activity (Online Resource 1) and phosphorylation at these sites has been associated with patient response to tamoxifen [6, 7]. In contrast, Beelen et al. showed an indication of tamoxifen resistance in postmenopausal breast cancer patients with activated MAPK [8]. No studies to date assessed potential associations of phosphorylation of ERα, MAPK or AKT in patients who received both tamoxifen and aromatase inhibitor treatment, and how this compares to tamoxifen alone. To this end, these phospho-modifications as potential biomarkers for selective endocrine therapy benefit were tested, as determined in the IES study. Additionally, immunohistochemistry (IHC) for active MAPK (phosphorylated at threonine residues 202 and 204) as well as AKT (phosphorylated at threonine 308 and serine 473) was undertaken, since these kinases are known to phosphorylate ERα. Specifically, MAPK phosphorylates S118ERα [9, 10], while AKT stimulates the phosphorylation of S167ERα [11]. Although reports using phospho-specific antibodies have indicated that these post-translational modifications can have an impact on patient’s outcome after adjuvant endocrine treatment [6, 7], none of these factors has been tested for biomarker potential in the context of a randomised clinical trial, directly comparing outcome after sequential tamoxifen/AI or tamoxifen alone.

Our hypothesis was that activated MAPK and/or AKT pathways—and their downstream impact on ERα phosphorylation at S118 and S167—might be predictive of differential treatment benefit of patients who were treated with tamoxifen alone or who received tamoxifen/exemestane switched therapy.

Our aims in this study were therefore three-fold: firstly, to assess the prognostic significance of the ERα phosphorylation markers in the entire study cohort regardless of treatment received. Secondly, to determine the correlations of the ERα phosphorylation with the respective kinases. Lastly, we aimed to determine whether these markers would indicate selective treatment benefit for patients receiving either tamoxifen alone or for those patients who switched to an AI after 2–3 years of tamoxifen.

## Methods

### Patients, data handling and sample collection

The study design, detailed eligibility criteria and treatment schedules have been previously described [2]. IES was a multicentre, international, randomised, double-blind phase III study, comparing exemestane 25 mg/day to tamoxifen 20 mg/day (30 mg in Denmark) prescribed for 2–3 years in postmenopausal women with ER+/unknown primary breast cancer who remained disease free after receiving adjuvant tamoxifen therapy for 2 to 3 years [4]. The IES study recruited in total 4724 postmenopausal women from 37 countries (366 centres) between 1998 and 2003 [4]. Formalin-fixed paraffin-embedded (FFPE) tumour samples were retrospectively collected from a subset of centres (PathIES centres *N* = 89) in accordance with institutional guidelines, ethics requirements and national laws. Of 1506 IES patients managed by PathIES centres, pathological samples from the primary surgery (at least 2 years before randomisation) were collected retrospectively from 1282 women recruited in PathIES centres (85.1%) [4].

All clinical data used in the analyses were based on the snapshot taken for the most recent IES clinical publication (median follow-up time was 91 months) [12] and the REMARK criteria were employed for data reporting [13].

### Immunohistochemistry staining

Tissue microarrays (TMAs) were constructed using formalin-fixed paraffin-embedded (FFPE) tumour blocks with a total of two cores per tumour. For details on antibodies, staining and scoring, see Online Resource methods section.

### Statistical analyses

Spearman’s correlation coefficients (*r*_S_) were obtained to investigate the associations between the continuous variables of phospho-markers (pT202/T204MAPK, pS118ERα and pS167ERα) and ERα, PR and Ki67. Trend test was used to assess association for ordinal variables (HER2 status, pT308AKT, pT473AKT and other dichotomised phospho-markers). Chi-squared (*χ*^2^) test was applied to investigate the association between the baseline characteristics of participants who did and did not provide tumour samples within PathIES participating centres. Disease-free survival (DFS) was defined as time from randomisation to recurrence (local, distant ipsilateral or contralateral) or death without disease relapse (intercurrent death) or censoring to the last date the patient was known to be alive and event free. Overall survival (OS) was defined as time from randomisation to date of death or censoring to the last date the patient was known to be alive.

The distributions of DFS and OS according to the subgroups of the phospho-markers were estimated using Kaplan–Meier plots censored at 10 years. Univariate and multivariable Cox proportional hazard (PH) survival models were applied to estimate hazard ratios (HR) for DFS and OS. All univariate and multivariable models met the PH assumption investigated with Schoenfeld residuals and PH tests.

For each of the phospho-markers (pT308AKT, pT473AKT, pT202/T204MAPK, pS118ERα and pS167ERα), a CoxPH regression model was fitted in the whole study, regardless of treatment received to assess the prognostic effect on DFS and OS via estimation of hazard ratios and 95% confidence intervals (CI). CoxPH models were fitted with and without adjusting for pre-specified prognostic factors of the centrally assessed estrogen-receptor status (H score), progesterone-receptor status (*H* score), Ki67 (ln(ki67 + 0.1)), HER2 status, treatment (tamoxifen and exemestane), nodal status, age group, tumour grade and size (ln(size)). Missing values of the clinicopathological variables were assumed as missing at completely random and therefore not imputed. In the multivariable survival modelling, interaction tests were used to investigate whether there is a differential treatment effect within phospho-marker-defined subgroups.

*P*-values for all statistical tests were two sided and Benjamini–Hochberg adjusted for multiple testing with false discovery rate of 10%. If the Benjamini–Hochberg adjusted *P*-value (*p*_BH_) was less than 0.05, the test was considered statistically significant.

## Results

### PathIES participants

**Fig. 1:**
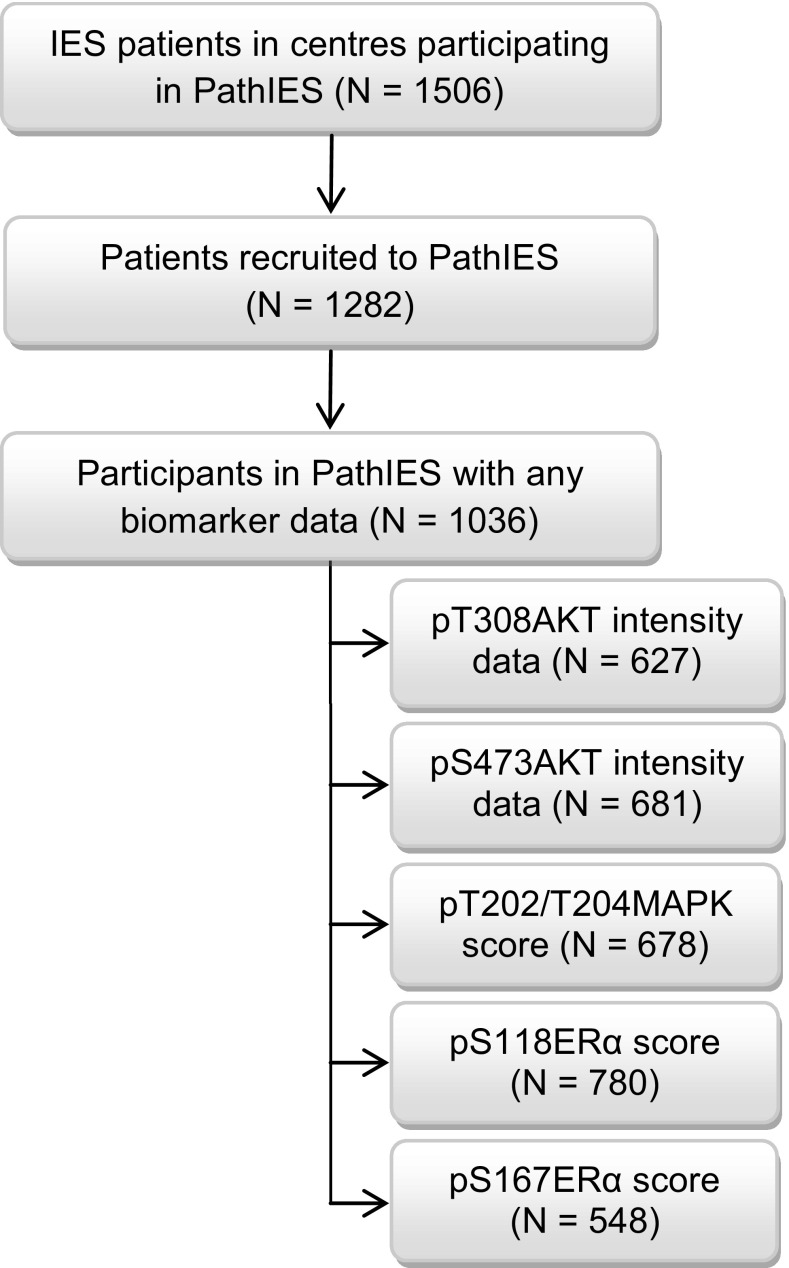
PathIES participants. Flow chart for PathIES participants with phospho-marker data

Of the 4724 postmenopausal women with ERα-positive/unknown primary breast cancer in IES trial, 1506 were managed in 89 centres participating in PathIES study (Fig. 1; Table 1). These centres recruited 1282 (85%, 1282/1506) women into PathIES of whom 1036 had phospho-marker data (Fig. 1; Table 1 and Online Resource 4).

**Table 1:** Baseline characteristics of PathIES participants

	PathIES Centres provided tissues (*N* = 1506)						Centres not provided tissues	
	Participants with any BM scores		*χ*^2^ test within centre, *p*	Participants without tissues/any BM scores		*χ*^2^ test with and without tissue provided, *p*	Participants without tissue/any BM scores	
	Total *N* = 1036			Total *N* = 470			Total *N* = 3218	
	*N*	%		*N*	%		*N*	%
Treatment								
A—exemestane	534	51.5		224	47.7		1594	49.5
B—tamoxifen	502	48.5		246	52.3		1624	50.5
			0.16					
						0.20		
Age (years)								
< 60	347	33.5		145	30.9		1031	32.0
60–69	452	43.6		220	46.8		1349	41.9
70 +	237	22.9		105	22.3		838	26.0
			0.48					
						0.20		
Grade (G)								
G1	186	18.0		86	18.3		517	16.1
G2	453	43.7		180	38.3		1354	42.1
G3/undifferentiated	199	19.2		79	16.8		645	20.0
Not assessable	10	1.0		17	3.6		76	2.4
Unknown	188	18.1		108	23.0		626	19.5
			0.60^a^					
						0.60		
Nodes (*N*)								
N−	447	43.1		229	48.7		1171	55.0
1–3 *N* +	371	35.8		149	31.7		911	28.3
> 3 *N* +	159	15.3		55	11.7		444	13.8
Unavailable	59	5.7		37	7.9		92	2.9
			0.03					
						< 0.001		
Tumour size (cm)								
≤ 2	596	57.5		290	61.7		1899	59.0
> 2 and ≤ 5	393	37.9		152	32.3		1171	36.4
> 5	31	3.0		7	1.5		84	2.6
Unavailable	16	1.6		21	4.5		64	2.0
			0.04					
						0.33		
Histology type								
Infiltrating ductal	768	74.1		336	71.5		2503	77.8
Infiltrating lobular	160	15.5		65	13.8		437	13.6
Other	108	10.4		69	14.7		269	8.4
Unavailable	0	0		0	0		9	0.2
			0.05					
						0.13		
Previous CT use								
No	839	81.0		364	77.4		1979	61.5
Yes	197	19.0		106	22.6		1239	38.5
			0.11					
						< 0.001		
HRT use								
No	677	65.3		111	23.6		690	21.4
Yes	323	31.2		333	70.9		2477	77.0
Unknown	36	3.5		26	5.5		51	1.6
			0.005					
						< 0.001		

Comparison of patient’s baseline characteristics who did and did not provide tumour samples within PathIES participating centres

*BM* biomarker, *CT* chemotherapy, *HRT* hormonal replacement therapy

^a^χ^2^ test includes G1, G2 and G3/undifferentiated groups only

### Staining and scoring of the phospho-markers

**Fig. 2:**
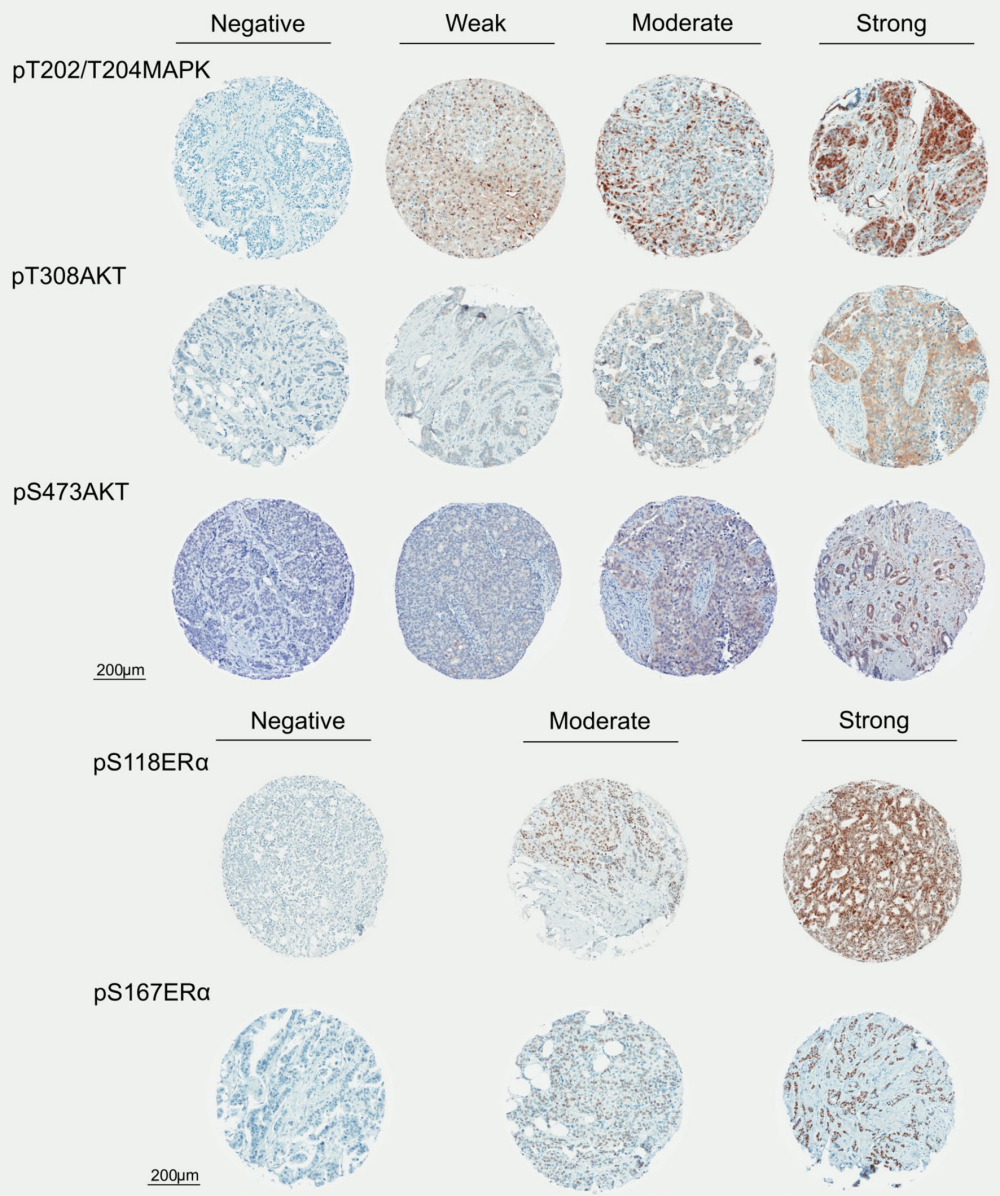
Immunostaining panel, depicting representative TMA cores. Representative images of immunostaining for each phospho-marker (pT202/T204MAPK, pT308AKT, pS473AKT, pS118ERα and pS167ERα) with range of intensity

Representative images of immunostaining for each marker with range of intensity are shown in Fig. 2. Good agreement was found between the independent observers when assessing the expression levels of the phospho-markers (Online Resource 5). Phospho-T308AKT, pS473AKT, pT202/T204MAPK and pS167ERα were detectable in 47.4% (297/627), 51.1% (348/681), 46.8% (316/675) and 52.7% (329/624) of the tumour samples, respectively (Table 2, Online Resource 6, 7). 51.3% (400/780) of the patients had pS118ERα of 0–40% and 48.7% (380/780) presented pS118ERα of ≥ 50% (Table 2, Online Resource 6, 7). Previous studies regarding pT202/T204MAPK, pS118ERα and/or pS167ERα often made use of a negative versus positive cut-off comparison [14–17], a cut-off point we also used for our pT202/T204MAPK and pS167ERα stainings. For the pS118ERα, however, we used a median based cut-off, yielding well-balanced groups by treatments (Table 2). Additionally, this approach allowed us to prevent the risk of any spuriously significant result associated with the use of optimal cut-off points [18, 19].

**Table 2:** Staining results of phospho-markers

Phospho-markers	Total	Tamoxifen	Exemestane	Test for trend
	*N*	*N* (%)	*N* (%)	*p*_BH_
pT308AKT (*N* = 627)				
No intensity	330	155 (51.7)	175 (53.5)	0.78
With intensity	297	145 (48.3)	152 (46.5)	
pS473AKT (*N* = 681)				
No intensity	333	160 (48.3)	173 (52.4)	0.78
With intensity	348	171 (51.7)	177 (47.6)	
pT202/T204MAPK (%) (*N* = 675)				
0	359	160 (49.8)	199 (56.2)	0.40
≥ 10	316	161 (50.2)	155 (43.8)	
pS118ERα (%) (*N* = 780)				
0–40	400	185 (49.2)	215 (53.2)	0.43
≥ 50	380	191 (50.8)	189 (46.8)	
pS167ERα (%) (*N* = 624)				
0	295	133 (44.6)	162 (49.7)	0.43
≥ 10	329	165 (55.4)	164 (50.3)	

Distribution of the phospho-markers by treatment strategies and the associated trend tests

*p*_BH_ Benjamini–Hochberg adjusted *p*

### Correlations between phospho-markers and clinical variables

As MAPK and AKT signalling cascades are functionally implicated in phosphorylation events on ERα, we next tested correlations between all phospho-markers of interest. All phospho-markers of MAPK and ERα (pT202/T204MAPK, pS167ERα and pS118ERα) are positively correlated, albeit moderately [Spearman’s correlation coefficients *r*_S_ (pT202/T204MAPK/pS118ERα) = 0.62, *r*_S_ (pT202/T204MAPK/pS167ERα) = 0.58, *r*_S_ (pS167ERα/pS118ERα) = 0.59], yet highly statistically significant (*p*_BH_ = 0.0002 for all) (Table 3).

**Table 3:** Positive correlation of pT202/T204MAPK, pS167ERα and pS118ERα

	pT202/T204 MAPK (%)	pS118ERα (%)	pS167ERα (%)
pS118ERα (%)			
n^a^	608	–	–
r_S_^b^	0.62	–	–
p_BH_^c^	0.0002		
pS167ERα (%)			
n	571	582	–
r_S_	0.58	0.59	–
p_BH_	0.0002	0.0002	
ER (*H* score)			
n	596	678	540
r_S_	0.17	0.25	0.33
p_BH_	0.0002	0.0002	0.0002
PR (*H* score)			
n	563	670	528
r_S_	0.12	0.17	0.12
p_BH_	0.005	0.0002	0.005
Ki67 (cont.)			
n	499	583	461
r_S_	0.01	0.01	0.10
p_BH_	0.79	0.79	0.05

Spearman’s correlation of the phospho-markers and prognostic factors

*cont*. continuous

^a^n—sample size

^b^r_S_—Spearman’s correlation coefficient

^c^p_BH_—Benjamini–Hochberg adjusted *p*

Furthermore, phosphorylation status of both pT308AKT and pS473AKT was associated with high levels of pT202/T204MAPK, pS167ERα and pS118ERα (*p*_BH_ < 0.001 for all) (Table 4). Similarly, a positive trend was found when comparing pT308AKT and pS473AKT (Table 4). These findings support the known biological connections between ERα phosphorylation status and activity of MAPK and AKT.

**Table 4:** Positive correlation of AKT activation with increased phosphorylation levels of MAPK and ERα

	pT308AKT				pS473AKT			
	Total	No int.	Int.	Test for trend	Total	No int.	Int.	Test for trend
	*N*	*N* (%)	*N* (%)	*p*_BH_	*N*	*N* (%)	*N* (%)	*p*_BH_
pT202/T204 MAPK (%)								
0	292	203 (69)	89 (33)	< 0.001	321	195 (68)	126 (41)	< 0.001
≥ 10	271	90 (31)	181 (67)		273	93 (32)	180 (59)	
pS118ERα (%)								
0–40	292	206 (67)	86 (31)	< 0.001	320	195 (64)	125 (39)	< 0.001
≥ 50	290	102 (33)	188 (69)		306	112 (36)	194 (61)	
pS167ERα (%)								
0	253	169 (61)	84 (32)	< 0.001	262	148 (56)	114 (38)	< 0.001
≥ 10	287	110 (39)	177 (68)		299	114 (44)	185 (62)	
pS473AKT								
No intensity	269	187 (62)	82 (31)	< 0.001	–	–	–	–
Intensity	298	115 (38)	183 (69)		–	–	–	

Distribution of pT202/T204MAPK, pS118ERα and pS167ERα by the groups of phosphorylated AKT and the associated trend tests

*int*. intensity, *p*_BH_ Benjamini–Hochberg adjusted *p*

The Spearman’s correlation of pT202/T204MAPK, pS167ERα and pS118ERα with PR status and Ki67 was overall negligible (Table 3). Exploring the distribution of dichotomised phospho-markers by HER2 status, we found more patients with pT308AKT (71%, *p*_BH_ = 0.03) or pS473AKT intensity (69%, *p*_BH_ = 0.06) in the HER2-positive group (Table 5).

**Table 5:** Association of phospho-markers with HER2 status

	HER2			Test for trend
	Total	Negative	Positive	
	*N*	*N* (%)	*N* (%)	*p*_BH_
pT308AKT				
No intensity	224	214 (55)	10 (29)	0.03
Intensity	202	178 (45)	24 (71)	
pS473AKT				
No intensity	215	203 (50)	12 (31)	0.06
Intensity	230	203 (50)	27 (69)	
pT202/T204MAPK (%)				
0	257	230 (55)	27 (64)	0.30
≥ 10	205	190 (45)	15 (36)	
pS118ERα (%)				
0–40	290	260 (53)	30 (68)	0.09
≥ 50	244	230 (47)	14 (32)	
pS167ERα (%)				
0	217	195 (50)	22 (54)	0.62
≥ 10	217	198 (50)	19 (46)	

Distribution of the dichotomised phospho-markers by HER2 and the associated trend tests

*p*_BH_ Benjamini–Hochberg adjusted *p*

The distribution of the dichotomised phospho-markers among the groups of clinical and pathological characteristics is summarised in Online Resource 8, demonstrating that patients with high pT202/T204MAPK (≥ 10%), or pS118ERα (≥ 50%) present with lower grade tumours [p_BH_ (pT202/T204MAPK) = 0.01, *p*_BH_ (pS118ERα) = 0.05) and smaller tumour size (*p*_BH_ (pT202/T204MAPK) = 0.01, *p*_BH_ (pS118ERα) = 0.01). Similarly, patients with high pS167ERα (≥ 10%) seemed to have smaller tumours (*p*_BH_ (pS167ERα) = 0.03]. Finally, a negative trend was observed between age and pT202/T204MAPK as well as pS118ERα; however, these trends were not statistically significant at 10% false discovery rate: older patients tend to have lower phosphorylation levels of MAPK (*p*_BH_ = 0.07) and ERα-S118 (*p*_BH_ = 0.07) (Online Resource 8).

### Associations of phospho-markers with DFS and OS outcomes

**Fig. 3:**
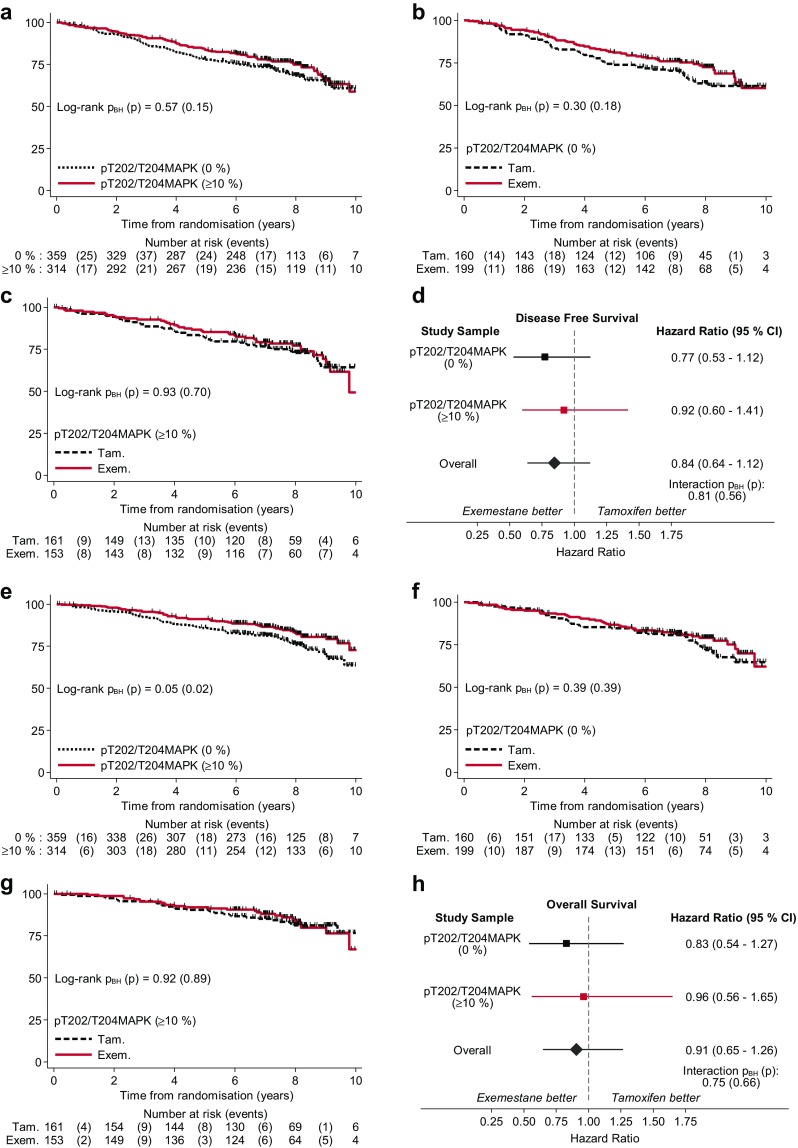
Kaplan–Meier DFS and OS estimates for pT202/T204MAPK. **a** DFS and **e** OS estimates by pT202/T204MAPK groups regardless of treatments received. **b** DFS and **f** OS estimates by treatments for patients with pT202/T204MAPK of 0%. **c** DFS and **g** OS estimates by treatments for patients with pT202/T204MAPK intensity of ≥ 10%. Forest plots represent the treatment effects of exemestane versus tamoxifen on **d** DFS and **h** OS in the subgroups of pT202/T204MAPK as well as in the whole study sample (overall). Hazard ratios were estimated with univariate CoxPH models. Test for interaction between exemestane versus tamoxifen and pT202/T204MAPK of ≥ 10% versus 0% is shown in the forest plots. (*p* unadjusted, *p*_*BH*_ Benjamini–Hochberg adjusted, *Tam* tamoxifen, *Exem* exmestane)

**Fig. 4:**
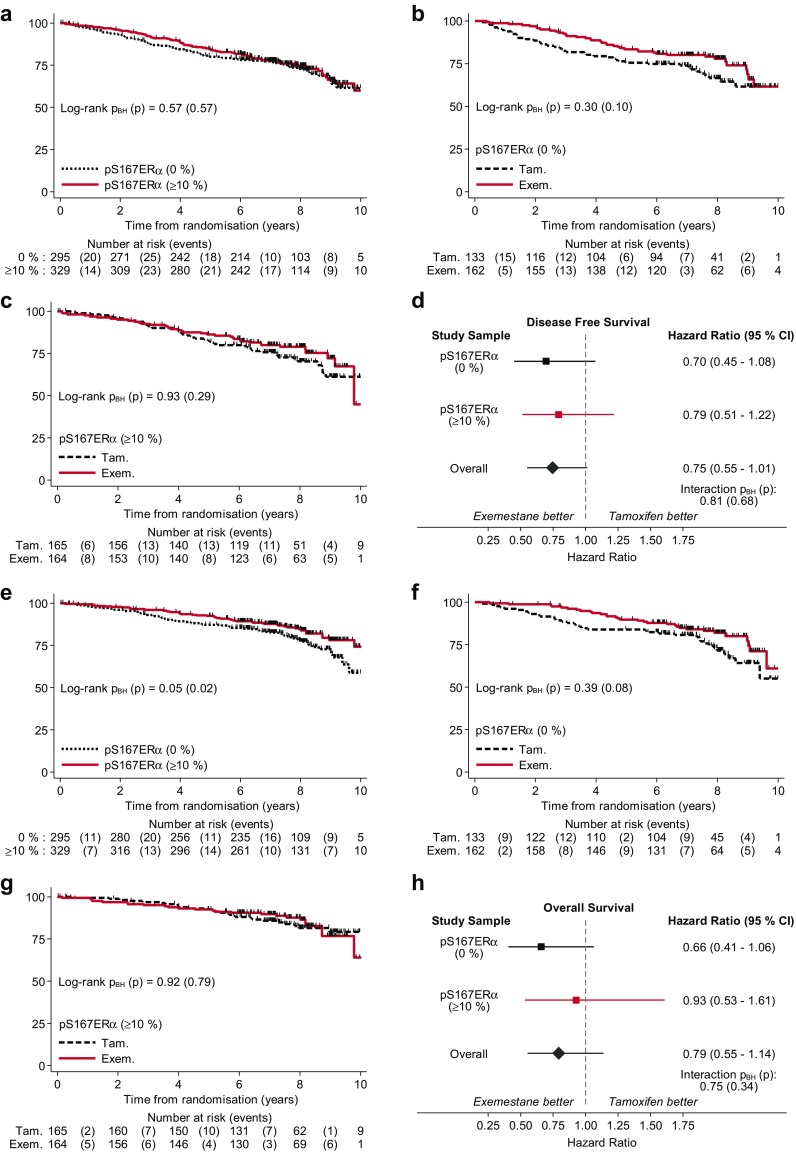
Kaplan–Meier DFS and OS estimates for pS167ERα. **a** DFS and **e** OS estimates by pS167ERα groups regardless of treatments received. **b** DFS and **f** OS estimates by treatments for patients with pS167ERα of 0%. **c** DFS and **g** OS estimates by treatments for patients with pS167ERα intensity of ≥ 10%. Forest plots represent the treatment effects of exemestane versus tamoxifen on **d** DFS and **h** OS in the subgroups of pT202/T204MAPK as well as in the whole study sample (overall). Hazard ratios were estimated with univariate CoxPH models. Test for interaction between exemestane versus tamoxifen and pS167ERα of ≥ 10% versus 0% is shown in the forest plots. (*p* unadjusted, *p*_*BH*_ Benjamini–Hochberg adjusted, *Tam* tamoxifen, *Exem* exmestane)

The potential associations of pS118ERα, pS167ERα, pT202/T204MAPK, pT308AKT and pS473AKT with outcome, and their relation to therapy were explored. Firstly, Kaplan–Meier estimates for DFS as primary endpoint for IES were analysed for all patients irrespective of therapy. No statistically significant difference in DFS estimates was observed for any of the factors tested (log-rank *p*_BH_ > 0.05) (Figs. 3, 4, Online Resource 9, 10, 11). When investigating how patients with different levels of phospho-markers would respond to tamoxifen and to switched therapy, no statistically significant change in the Kaplan–Meier curves for DFS was revealed for any biomarkers.

The effects of the phosphorylation levels of the markers on overall survival were also explored with Kaplan–Meier curves (Figs. 3, 4, Online Resource 9, 10, 11). Phosphorylation levels of the biomarkers were not statistically significantly associated with the overall survival outcome of the PathIES participants. Patients with higher levels of pT202/T204MAPK (≥ 10%) or pS167ERα (≥ 10%) tend to have better OS than those with pT202/T204MAPK of 0% (log-rank *p*_BH_ = 0.05) (Fig. 3e) or pS167ERα of 0% (log-rank *p*_BH_ = 0.05) (Fig. 4e); however, none of these associations were statistically significant at 10% false discovery rate. The association of the levels of the phospho-markers with DFS was next tested in the whole PathIES study sample with CoxPH survival models. None of the phospho-markers was found to be prognostic for DFS either in the univariate or in the multivariable CoxPH models adjusting the effect of each phospho-marker for the prognostic parameters of ERα, PR, HER2, Ki67, tumour size and grade, nodal status, age and treatment regimens (Table 6).

**Table 6:** Association of the phospho-markers with disease-free survival (DFS)

Phospho-markers	Univariate CoxPH		Multivariable CoxPH^a^		
	HR (95% CI)	p_BH_	HR (95% CI)	p_BH_	Int.^b^ p_BH_
pT308AKT					
No intensity	1.00		1.00		
With intensity	0.89 (0.66–1.21)	0.57	1.26 (0.68–2.35)	0.90	0.90
pS473AKT					
No intensity	1.00		1.00		
With intensity	1.09 (0.89–1.45)	0.57	0.94 (0.53–1.66)	0.90	0.90
pT202/T204MAPK (%)					
0	1.00		1.00		
≥ 10	0.81 (0.61–1.08)	0.57	0.88 (0.49–1.56)	0.90	0.90
pS118ERα (%)					
0–40	1.00		1.00		
≥ 50	0.86 (0.65–1.12)	0.57	0.65 (0.40–1.06)	0.72	0.90
pS167ERα (%)					
0	1.00		1.00		
≥ 10	0.92 (0.67–1.24)	0.57	0.96 (0.53–1.76)	0.90	0.90

Univariate and multivariable CoxPH analyses of phospho-markers with DFS

*CI* confidence intervals, *p*_*BH*_ Benjamini–Hochberg adjusted *p*

^a^Adjusted for ER, PR, HER2, Ki67, tumour size and grade, nodal status, age and treatment

^b^Interaction between biomarker and exemestane versus tamoxifen

When investigating the predictive value of the phospho-markers with high versus low expression levels on DFS for exemestane over tamoxifen in the entire study sample, none of the biomarkers’ expression was statistically significant to predict differential DFS benefit for patients who switched therapy over tamoxifen: the phospho-marker and treatment interaction tests were not statistically significant in the multivariable analyses (*p*_BH_ corresponding to the interaction test > 0.05 for all) (Table 6).

Exploring the effect of pT202/T204MAPK on OS in the entire cohort, the crude effect size of pT202/T204MAPK of ≥ 10% versus 0% was 0.66 (95% CI 0.47 to 0.94) (Table 7). This would suggest an overall survival benefit among patients with pT202/T204MAPK of ≥ 10%; however, this was not statistically significant after adjusting for multiple testing at 10% false discovery rate (*p*_BH_ = 0.06). The multivariable analyses further demonstrated that this slight association of pT202/T204MAPK with the OS was due to the confounding effect of conventional parameters (HR 0.67, 95% CI 0.33 to 1.34, *p*_BH_ = 0.29). Similarly, patients (regardless of treatment received) who expressed high level of pS167ERα seemed to have a better prognosis for OS than those with low expression of pS167ERα but this association was not statistically significant (crude HR 0.66, 95% CI 0.46 to 0.94, *p*_BH_ = 0.06; adjusted HR 0.58, 95% CI 0.27 to 1.26, *p*_BH_ = 0.29) (Table 7). The other markers (pS118ERα, pT308AKT and pT473AKT) were not prognostic for OS in either univariate or multivariable analyses (Table 7).

**Table 7:** Association of the phospho-markers with overall survival (OS)

Phospho-markers	Univariate CoxPH		Multivariable CoxPH^a^		
	HR (95% CI)	*p*_BH_	HR (95% CI)	p_BH_	Int.^b^*p*_BH_
pT308AKT					
No intensity	1.00		1.00		
With intensity	0.73 (0.50–1.06)	0.16	1.55 (0.74–3.25)	0.29	0.77
pS473AKT					
No intensity	1.00		1.00		
With intensity	0.93 (0.66–1.30)	0.66	0.69 (0.35–1.37)	0.29	0.96
pT202/T204MAPK (%)					
0	1.00		1.00		
≥ 10	0.66 (0.47–0.94)	0.06	0.67 (0.33–1.34)	0.29	0.77
pS118ERα (%)					
0–40	1.00		1.00		
≥ 50	0.83 (0.60–1.14)	0.30	0.50 (0.27–0.93)	0.14	0.96
pS167ERα (%)					
0	1.00		1.00		
≥ 10	0.66 (0.46–0.94)	0.06	0.58 (0.27–1.26)	0.29	0.82

Univariate and multivariable CoxPH analyses of phospho-markers with OS

*CI* confidence intervals, *p*_*BH*_ Benjamini–Hochberg adjusted *p*

^a^Adjusted for ER, PR, HER2, Ki67, tumour size and grade, nodal status, age and treatment

^b^Interaction between biomarker and exemestane versus tamoxifen

Interaction tests showed no differential treatment (exemestane over tamoxifen) effect on OS within any of the phospho-markers-defined subgroups (*p*_BH_ > 0.05 for all) (Table 7).

In post hoc exploratory analyses of the combinations of factors within the same biological pathway (pT202/T204MAPK/pS118ERα, pS473AKT/pS167ERα and pT308AKT/pS167ERα), there were no differences observed in DFS (Online Resource 12, 13) or OS (Online Resource 12, 14) outcomes for any of the tested combinations.

Interaction tests between the phospho-markers and treatments demonstrated no predictive value of any pathways investigated either on DFS or on OS among patients treated with exemestane over tamoxifen when adjusting for potential confounders in the entire study sample (all *p*_BH_ values corresponding to the interaction test > 0.05).

## Discussion

In the adjuvant treatment of breast cancer, multiple endocrine therapeutic options are available and current guidelines permit the use of tamoxifen, aromatase inhibitors or a sequential treatment of the two. Therefore, biomarkers are needed to enable optimal endocrine treatment selection. In this study, we used samples from the Intergroup Exemestane Study to evaluate whether there is predictive value of biomarkers in the MAPK/AKT/ERα signalling axis selective for patients receiving either tamoxifen monotherapy or tamoxifen/exemestane sequential treatment. While multiple studies have described an association between tamoxifen response and phosphorylation status of these factors [6–8], such connections are thus far not reported in patients who received both tamoxifen and aromatase inhibitor treatment.

Several studies [14, 20–22], including our own [23–25], have evaluated co-expression of relevant MAPK and AKT pathways with kinases with ERα phosphorylation status; in general, these studies have reported a correlation between pS118ERα, pS167ERα and the activation status of respective kinases, i.e. MAPK and AKT. Our current study confirms these findings, further supporting the quality of our dataset.

In the context of PathIES study, the phospho-markers of our interest did not appear to be prognostic for DFS in the entire cohort regardless of treatment received or predictive for this outcome among patients with switched therapy (to exemestane from tamoxifen), over those treated with tamoxifen alone when adjusting for potential confounders.

Phospho-S167ERα has previously been shown to be positively correlated with PR [26] and, by our group, negatively with tumour size [24]. Although it has been reported that pS167ERα is indicative of good outcome in patients who received adjuvant tamoxifen [24, 26, 27], the present study demonstrated that this biomarker is neither prognostic for DFS or OS nor predictive for these outcomes among PathIES patients managed with exemestane after tamoxifen when controlling for conventional prognostic factors.

In terms of effect on prognosis, several studies have been published examining the effect of pS118ERα where this marker correlates with PR [28] and is negatively correlated with grade [25]. As the association of pS118ERα with outcome is most profound in pre-menopausal patients [16], any potential inconsistency of our findings with previous reports may be related to differences in menopausal status. Furthermore, our group has previously shown an association between pT202/T204MAPK and smaller tumour size, and better survival outcome in ERα-positive breast cancer patients [24]. The present study appears to confirm the negative associations of both factors (pT202/T204MAPK and pS118ERα) with prognostic features such as tumour size, yet no significant association with outcomes was observed in this cohort for either phosphorylation marker.

Activation of the phosphatidyl-inositol-3 kinase pathway as measured by phosphorylation status of components of the protein cascade has been shown to correlate with tamoxifen resistance, while this was not found for its upstream drivers like the presence of a PIK3CA hotspot mutation, or PTEN loss [29, 30]. AKT inhibitors have been shown to extend the duration of response to both tamoxifen and AI in pre-clinical models [31]. It has also been reported that high AKT activity, as defined by phosphorylation at serine 473 and threonine 308, does not predict for significant benefit from tamoxifen [8]. In this study, the correlations between AKT phosphorylation and poor prognosis in ERα-positive patients were not observed, although high expression of its downstream target p-p70S6K had been reported to confer a favourable prognosis in postmenopausal patients [8]. Data in this study which supported the correlations with conventional prognostic factors, AKT phosphorylation, however, showed no independent impact on prognosis in this randomised phase III study population.

## Conclusion

This study of 1036 primary tumours confirms the association between activated AKT, MAPK and ERα phosphorylation status in postmenopausal breast cancer patient, but does not corroborate their prognostic power for DFS or OS in the entire PathIES study, nor their predictive values for these outcomes for patients managed by switched therapy over tamoxifen alone.

## Electronic supplementary material

Below is the link to the electronic supplementary material.

Supplementary material 1 (PDF 614 KB)

## Data Availability

The clinical dataset and IHC images analysed during the current study are not publicly available due to ethical legislation.
