# Supplementary material for: Dissecting the predictive value of MAPK/AKT/estrogen-receptor phosphorylation axis in primary breast cancer to treatment response for tamoxifen over exemestane: a Translational Report of the Intergroup Exemestane Study (IES)—PathIES
Source: Breast Cancer Res Treat. 2019 Jan 24;175(1):149–63. doi: 10.1007/s10549-018-05110-x (PMC6491661; doi:10.1007/s10549-018-05110-x)
Supplement: Supplementary file 1 — Supplementary material 1 (PDF 614 KB) [file 10549_2018_5110_MOESM1_ESM.pdf]

Breast Cancer Research and Treatment

Dissecting the predictive value of MAPK/AKT/Estrogen Receptor phosphorylation axis in primary breast cancer to treatment response for tamoxifen over exemestane: a Translational Report of The Intergroup Exemestane Study (IES) – PathIES

Zsolt Szijgyarto[1*], Koen D. Flach[2*], Mark Opdam[3], Carlo Palmieri[4,5,6], Sabine C. Linn[3,7,8], Jelle Wesseling[3,9], Simak Ali[6], Judith M Bliss[1], Maggie Chon U Cheang[1], Wilbert Zwart[2], R. Charles Coombes[6+]

[1]Clinical Trials and Statistics Unit (ICR-CTSU), Division of Clinical Studies, The Institute of Cancer Research, London, SM2 5NG, UK
[2]Division of Oncogenomics, Oncode Institute, the Netherlands Cancer Institute, 1066CX Amsterdam, the Netherlands
[3]Division of Molecular Pathology, the Netherlands Cancer Institute, Plesmanlaan 121, 1066CX Amsterdam, the Netherlands
[4]Department of Molecular and Clinical Cancer Medicine, University of Liverpool, Liverpool, L69 3BX, UK
[5]Academic Department of Medical Oncology, Clatterbridge Cancer Centre NHS Foundation Trust, Wirral, CH63 4JY, UK
[6]Department of Surgery and Cancer, Imperial College London, London, W12 0NN, UK
[7]Department of Medical Onology, the Netherlands Cancer Institute, Plesmanlaan 121, 1066CX Amsterdam, the Netherlands
[8]Department of Pathology, University Medical Center Utrecht, Utrecht, Netherlands
[9]Department of Pathology, the Netherlands Cancer Institute, Plesmanlaan 121, 1066CX Amsterdam, the Netherlands
*these authors contributed equally to this work

Authors' email addresses: Zsolt Szijgyarto: Zsolt.Szijgyarto@icr.ac.uk; Koen D. Flach: k.flach@nki.nl; Mark Opdam: m.opdam@nki.nl; Carlo Palmieri: C.Palmieri@liverpool.ac.uk; Sabine Linn: s.linn@nki.nl; Jelle Wesseling: j.wesseling@nki.nl; Simak Ali: simak.ali@imperial.ac.uk; Judith M Bliss: Judith.Bliss@icr.ac.uk; Maggie Chon U Cheang: Maggie.Cheang@icr.ac.uk; Wilbert Zwart: w.zwart@nki.nl; R. Charles Coombes: c.coombes@imperial.ac.uk

[+]**To whom correspondence should be addressed:** Professor Raoul Charles Coombes, Department of Cancer and Surgery, Faculty of Medicine, Imperial College London, Du Cane Road, London W12 0NN, UK, (+44) 0207 594 2135 c.coombes@imperial.ac.uk

Online Resource methods section

Immunohistochemistry staining

TMAs were stained for pT308AKT, pT202/T204MAPK, pS167ERα, pS118ERα and hematoxylin-eosin (HE) with the ULTRA BenchMark IHC/ISH Staining Module based at the Netherlands Cancer Institute Core Facility Molecular Pathology and Biobanking (Online Resource 2,3). Staining for pS473AKT was performed manually. Additionally, 23 whole tumour section slides were stained, with the exception of the pS118ERα staining for which 118 slides were used. The intensity (0 to 4) and percentage of staining-positive invasive tumour cells (0-100%) were scored by two independent observers. For each staining one TMA was used to calculate the inter-observer variability by using the weighted Cohen's kappa coefficient. When several slides were available for a given tumour, the highest score was used. The scores and/or the intensity of staining were provided for pT308AKT, pS473AKT, pT202/T204MAPK, pS167ERα and pS118ERα markers. There was no external reference cohort with defined thresholds for the phospho-markers investigated, therefore, data driven cut-offs were chosen at the beginning of the analyses to establish well-balanced subcategories: the pT202/T204MAPK, pS167ERα and pS118ERα scores were dichotomised as 0% versus ≥ 10%, 0% versus ≥ 10% and 0 - 40% versus ≥ 50%, respectively. Due to the small number of cases with pT308AKT and pS473AKT of medium and strong intensity, these markers were classified no intensity versus any intensity (weak/medium/strong). In addition, the phospho-markers were combined to assess activation of the pathways. Therefore, pS118ERα and pS167ERα H-scores were also calculated using the percentage and the intensity values from the IHC staining. Both phospho-markers were then dichotomised over their median (median H-score of pS118ERα: 100; median of pS167ERα: 50), giving subgroups characterised with their low and high expressions. The groups of low and high expression of pS118ERα and pS167ERα were combined with the low and high expression of pT202/T204MAPK as well as pAKT as follows: pT202/T204MAPK / pS118ERα, pT308AKT / pS167ERα and pT473AKT / pS167ERα with low/low, low/high, high/low and high/high expressions.

Online Resource 1 Schematic showing the functional domains and activation sites of ERα. ERα is activated through phosphorylation at serine residues 118 and 167 by MAPK and AKT

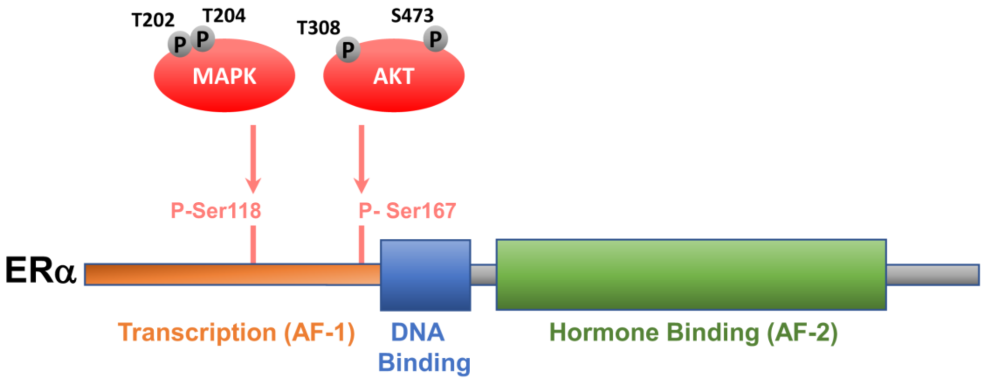

Online Resource 2 Antibodies used for immunostaining. Source and dilutions of antibodies applied for phospho-markers' staining

Staining	Antibody	Dilution
pT308AKT	Cell signaling #2965	1:50
pS473AKT	Cell signaling #4060	1:50
pT202/T204MAPK	Cell signaling #4370	1:400
pS167ERα	Cell signaling #5587	1:50
pS118ERα	Cell signaling #2511	1:1200

Online Resource 3 Immunostaining specifications. Antigen retrieval and antibody incubation time for each phospho-marker

Staining	Antigen retrieval incubation	Used buffer	Antibody incubation
pT308AKT	36 min	CC1[a]	1 hour
pS473AKT	15 min	Citrate	16 hours
pT202/T204MAPK	64 min	CC2[b]	1 hour
pS167ERα	44 min	CC2	1 hour
pS118ERα	92 min	CC1	0.5 hour

[a]CC1 (Ultra Cell Conditioning 1, Ventana, #950-224). [b]CC2 (Ultra Cell Conditioning 2, Ventana, #950-223)

Online Resource 4 PathIES participants with biomarker data. Comparison of patient's characteristics with and without any biomarker data (N=1036 versus 3688)

	Participants with any BM[a] scores Total N = 1036		Participants without any BM scores Total N = 3688		χ^2 test
	N	%	N	%	P-value
Treatment					0.20
Exemestane	534	51.5	1818	49.3	
Tamoxifen	502	48.5	1870	50.7	
Age (years)					0.20
<60	347	33.5	1176	31.9	
60-69	452	43.6	1569	42.5	
70+	237	22.9	943	25.6	
Grade (G)					0.60
G1	186	18.0	603	16.4	
G2	453	43.7	1534	41.6	
G3/Undifferentiated	199	19.2	724	19.6	
Not assessable	10	1.0	93	2.5	
Unknown	188	18.1	734	19.9	
Nodes (N)					<0.001
N-	447	43.1	2000	54.2	
1-3N+	371	35.8	1060	28.7	
>3N+	159	15.3	499	13.5	
Unavailable	59	5.7	129	3.5	
Tumour size (cm)					0.33
≤2	596	57.5	60.8	59.3	
>2 & ≤5	393	37.9	36.7	35.9	
>5	31	3.0	2.5	2.5	
Unavailable	16	1.6	85	2.3	
Histology type					0.13
Infiltrating ductal	768	74.1	2839	77.0	
Infiltrating lobular	160	15.5	502	13.6	
Other	108	10.4	338	9.2	
Unavailable	0	0	9	0.2	
Previous CT[b] use					<0.001
No	839	81.0	2343	63.5	
Yes	197	19.0	1345	36.5	
HRT[c] use					<0.001
No	677	65.3	2810	76.2	
Yes	323	31.2	801	21.7	
Unknown	36	3.5	77	2.1	

[a] BM – biomarker; [b]CT – chemotherapy; [c]HRT – hormone replacement therapy

Online Resource 5 Scoring of the phospho-markers by independent observers. Weighted Cohen's kappa coefficient for assessing inter-observer variability

**Staining**	**Cut-off**	**Kappa**
pT308AKT	Intensity *0 vs 1+2+3*	0.675
pS473AKT	Intensity *0 vs 1+2+3*	0.685
pT202/T204MAPK	Percentage *0% vs 10-100%*	0.916
pS167ERα	Percentage *0% vs 10-100%*	0.783
pS118ERα	Percentage *0-40% vs 50-100%*	0.823

Additional file 6 Staining scores of pT202/T204MAPK, pS118ERα and pS167ERα. Distribution of pT202/T204MAPK, pS118ERα pS167ERα by treatment strategies

	pT202/T204MAPK		pS118ERα		pS167ERα	
**Scores (%)**	**Tamoxifen N (%)**	**Exemestane N (%)**	**Tamoxifen N (%)**	**Exemestane N (%)**	**Tamoxifen N (%)**	**Exemestane N (%)**
0	160 (49.8)	199 (56.5)	41 (10.9)	57 (14.1)	133 (44.6)	162 (49.7)
10	52 (16.2)	46 (13.0)	34 (9.0)	49 (12.1)	48 (16.1)	49 (15.0)
20	13 (4.0)	15 (4.3)	57 (15.2)	51 (12.6)	17 (5.7)	21 (6.4)
30	20 (6.2)	12 (3.4)	36 (9.6)	29 (7.2)	17 (5.7)	14 (4.3)
40	14 (4.4)	15 (4.3)	17 (4.5)	29 (7.2)	13 (4.4)	13 (4.0)
50	17 (5.3)	15 (4.3)	18 (4.8)	25 (6.2)	12 (4.0)	14 (4.3)
60	5 (1.6)	8 (2.3)	26 (6.9)	29 (7.2)	6 (2.0)	5 (1.5)
70	8 (2.5)	11 (3.1)	33 (8.8)	28 (6.9)	15 (5.0)	10 (3.1)
80	8 (2.5)	9 (2.6)	27 (7.2)	33 (8.2)	7 (2.4)	10 (3.1)
90	9 (2.8)	10 (2.8)	56 (14.9)	40 (9.9)	15 (5.0)	14 (4.3)
100	15 (4.7)	12 (3.4)	31 (8.2)	34 (8.4)	15 (5.0)	14 (4.3)
Total	321 (100)	352 (100)	376 (100)	404 (100)	298 (100)	326 (100)

Online Resource 7 Histograms of staining scores (%) by treatments. **a** Scores of pT202/T204MAPK by treatments. **b** Scores of pS167ERα by treatments. **c** Scores of pS118ERα by treatments

a

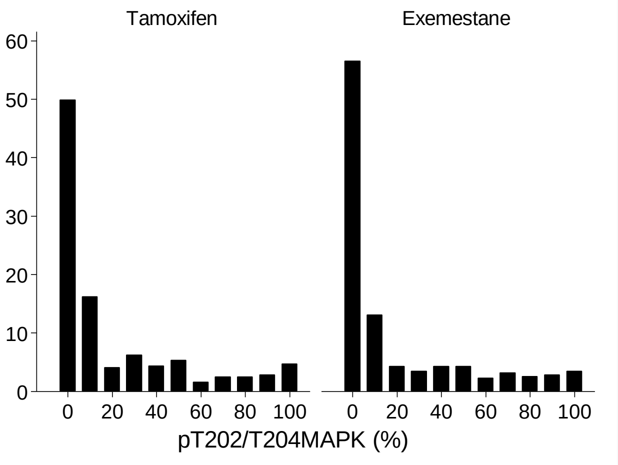

b

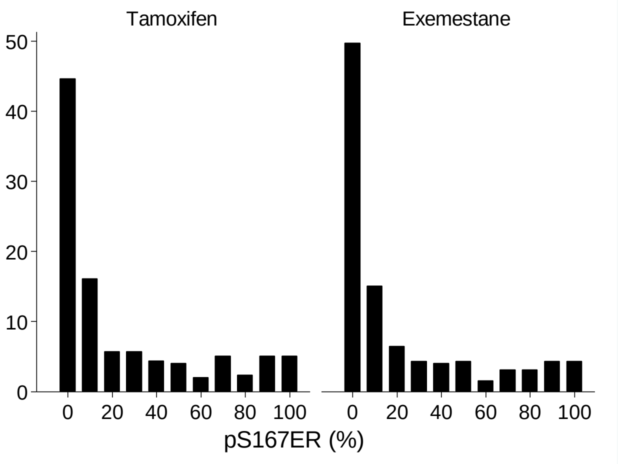

c

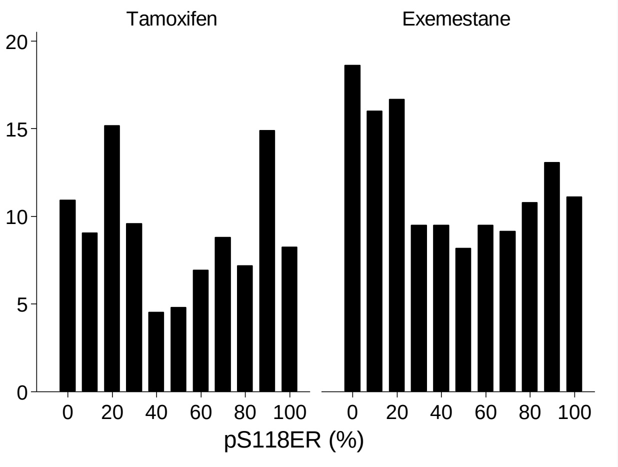

Online Resource 8 Association of the dichotomised phospho-markers with the clinical and pathological characteristics. Distribution of phospho-markers among the clinicopathological characteristics and the associated trend tests

	pT308AKT			pS473AKT			pT202/T204MAPK			pS118ERα			pS167ERα		
	**Total**	**no int.**[a]	**int.**	**Total**	**no int.**	**int.**	**Total**	**0%**	**≥10%**	**Total**	**0 - 40 %**	**≥50%**	**Total**	**0%**	**≥10%**
	**N**	**N (%)**	**N (%)**	**N**	**N (%)**	**N (%)**	**N**	**N (%)**	**N (%)**	**N**	**N (%)**	**N (%)**	**N**	**N (%)**	**N (%)**
**Age (years)**															
<60	219	108 (32.7)	111 (37.4)	234	111 (33.3)	123 (53.3)	227	107 (29.8)	120 (38.2)	270	123 (30.8)	147 (38.7)	206	88 (29.8)	118 (35.9)
60-69	268	150 (45.5)	118 (39.7)	297	151 (45.3)	146 (42.0)	292	162 (45.1)	130 (41.4)	330	176 (44.0)	154 (40.5)	274	134 (45.4)	140 (42.6)
70+	140	72 (21.8)	68 (22.9)	150	71 (21.3)	79 (22.7)	154	90 (25.1)	64 (20.4)	180	101 (25.3)	79 (20.8)	144	73 (24.7)	71 (21.6)
Trend test		p_{BH}[b] = 0.69			p_{BH} = 0.94			p_{BH} = 0.07			p_{BH} = 0.07			p_{BH} = 0.29	
**Hist. grade**															
G1	98	51 (18.0)	47 (18.7)	117	56 (20.4)	61 (20.6)	115	43 (14.7)	72 (27.1)	145	64 (19.3)	81 (24.8)	109	44 (17.6)	65 (23.6)
G2	305	171 (60.4)	134 (53.4)	302	148 (53.8)	154 (52.0)	300	163 (55.8)	137 (51.5)	353	173 (52.3)	180 (55.0)	287	141 (56.4)	146 (53.1)
G3	131	61 (21.6)	70 (27.9)	162	78 (25.8)	84 (27.4)	151	93 (29.5)	58 (21.4)	169	103 (28.4)	66 (20.2)	135	69 (26.0)	66 (23.3)
Trend test		p_{BH} = 0.42			p_{BH} = 0.94			p_{BH} = 0.01			p_{BH} = 0.05			p_{BH} =0.65	
**Tumour size (cm)**															
≤2	370	182 (55.7)	188 (64.0)	388	183 (55.8)	205 (59.4)	381	161 (45.5)	220 (70.5)	437	193 (49.0)	244 (65.2)	368	158 (51.4)	210 (64.8)
>2 & ≤5	229	129 (39.4)	100 (34.0)	262	133 (40.5)	129 (37.4)	262	178 (50.3)	84 (26.9)	306	185 (47.0)	121 (32.4)	227	121 (41.4)	106 (32.7)
>5	22	16 (4.9)	6 (2.0)	23	12 (3.7)	11 (3.2)	23	15 (4.2)	8 (2.6)	25	16 (4.0)	9 (2.4)	21	13 (4.5)	8 (2.5)
Trend test		p_{BH} = 0.07			p_{BH} = 0.53			p_{BH} = 0.01			p_{BH} = 0.01			p_{BH} = 0.03	
**Nodal status**															
negative	292	156 (50.0)	136 (48.9)	304	149 (47.0)	155 (47.7)	306	162 (47.0)	144 (49.7)	347	179 (47.6)	168 (46.4)	291	146 (52.3)	145 (47.2)
1-3 N+	204	105 (33.7)	99 (35.6)	234	115 (36.3)	119 (36.6)	226	127 (36.8)	99 (34.1)	277	137 (36.4)	140 (38.7)	200	88 (31.5)	112 (36.5)
4-9 N+	67	38 (12.2)	29 (10.4)	76	41 (12.9)	35 (10.8)	74	42 (12.2)	32 (11.0)	85	45 (12.0)	40 (11.0)	66	31 (11.1)	35 (11.4)
≥10 N+	27	13 (4.2)	14 (5.0)	28	12 (3.8)	16 (4.9)	29	14 (4.1)	15 (5.2)	29	15 (4.0)	14 (3.9)	29	14 (5.0)	15 (4.9)
Trend test		p_{BH} = 0.94			p_{BH} = 0.94			p_{BH} = 0.82			p_{BH} =0.94			p_{BH} = 0.53	
**Hist.[c] type**															
ductal	493	251 (76.1)	242 (81.5)	534	250 (75.1)	284 (81.6)	517	281 (78.3)	236 (75.2)	610	322 (80.5)	288 (75.8)	485	230 (78.0)	255 (77.5)
lobular	76	48 (14.5)	28 (9.4)	71	37 (11.1)	34 (9.8)	86	42 (11.7)	44 (14.0)	91	37 (9.3)	54 (14.2)	72	31 (10.5)	41 (12.5)
other	58	31 (9.4)	27 (9.1)	76	46 (13.8)	30 (8.6)	70	36 (10.0)	34 (10.8)	79	41 (10.3)	38 (10.0)	67	34 (11.5)	33 (10.0)
Trend test		p_{BH} = 0.30			p_{BH} = 0.08			p_{BH} = 0.53			p_{BH} = 0.34			p_{BH} = 0.98	

[a]int.- intensity; [b]p_{BH} - Benjamini-Hochberg adjusted p value, [c]Hist. - Histology

Online Resource 9 Kaplan-Meier DFS and OS estimates for pS118ERα. **a** DFS and **d** OS estimates by pS118ERα groups regardless treatments received. **b** DFS and **e** OS estimates by treatments for patients with pS118ERα intensity of 0 - 40%. **c** DFS and **f** OS estimates by treatments for patients with pS118ERα intensity of ≥ 50%. (Abbreviations: p – unadjusted, p_{BH} – Benjamini-Hochberg adjusted, Tam – tamoxifen, Exem – exmestane)

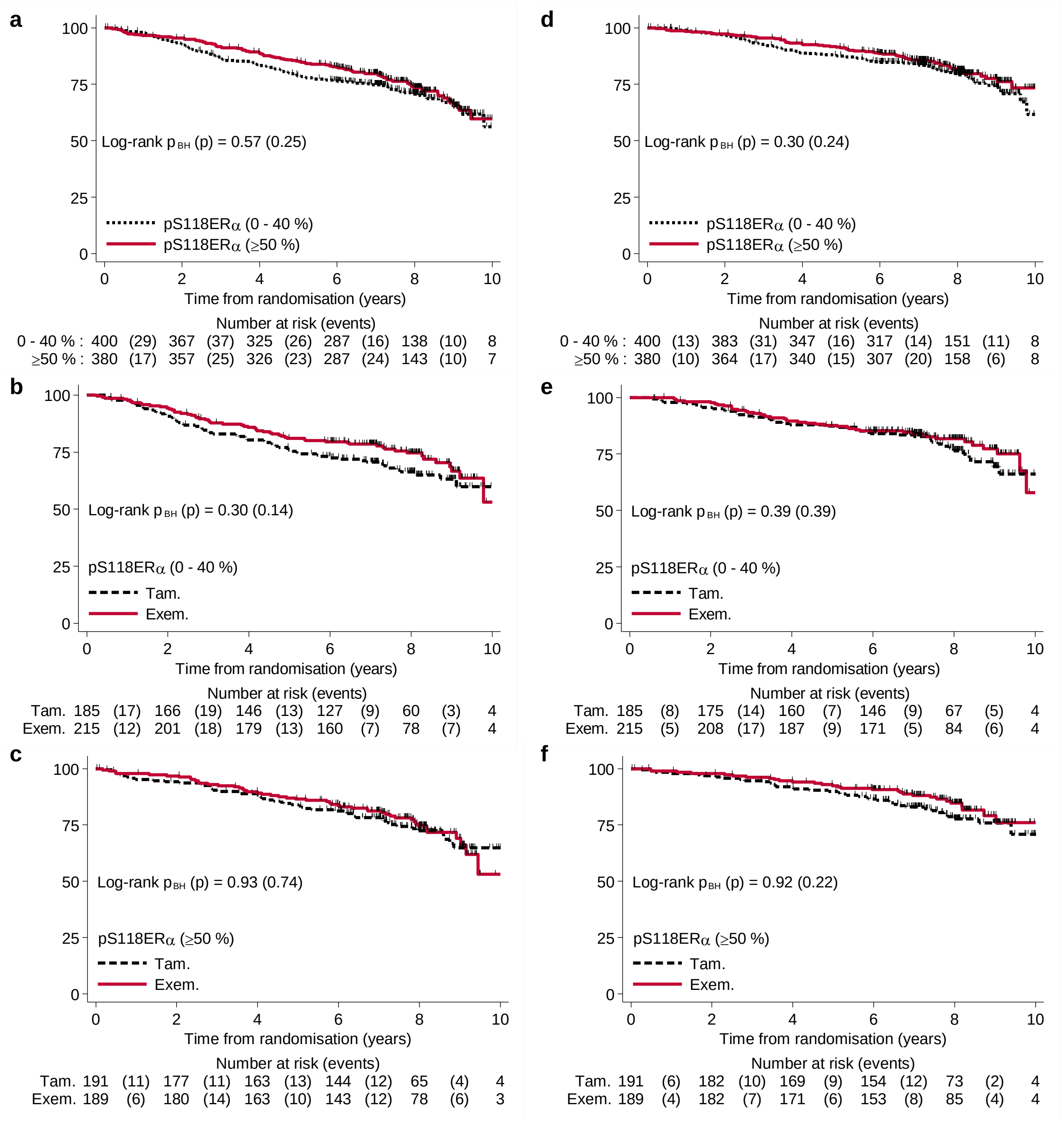

Online Resource 10 Kaplan-Meier DFS and OS estimates for pT308AKT **a** DFS and **d** OS estimates by pT308AKT groups regardless treatments received. **b** DFS and **e** OS estimates by treatments for patients without pT308AKT intensity. **c** DFS and **f** OS estimates by treatments for patients with pT308AKT intensity. (Abbreviations: p – unadjusted, p_{BH} – Benjamini-Hochberg adjusted, Tam – tamoxifen, Exem – exmestane)

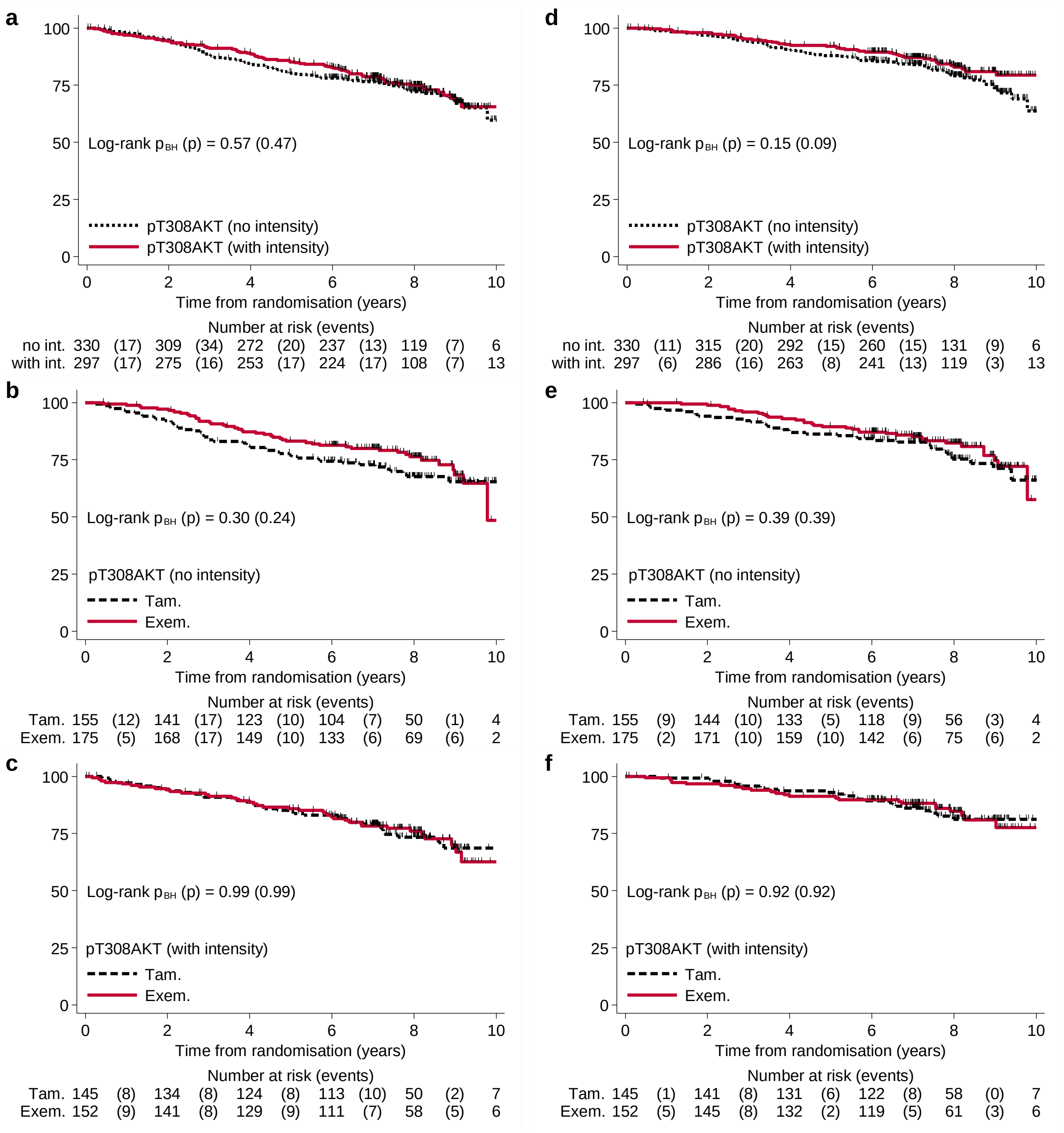

Online Resource 11 Kaplan-Meier DFS and OS estimates for pS473AKT. **a** DFS and **d** OS estimates by groups of pS473AKT regardless treatments received. **b** DFS and **e** OS estimates by treatments for patients without pS473AKT intensity. **c** DFS and **f** OS estimates by treatments for patients with pS473AKT intensity. (Abbreviations: p – unadjusted, p_{BH} – Benjamini-Hochberg adjusted, Tam – tamoxifen, Exem – exmestane)

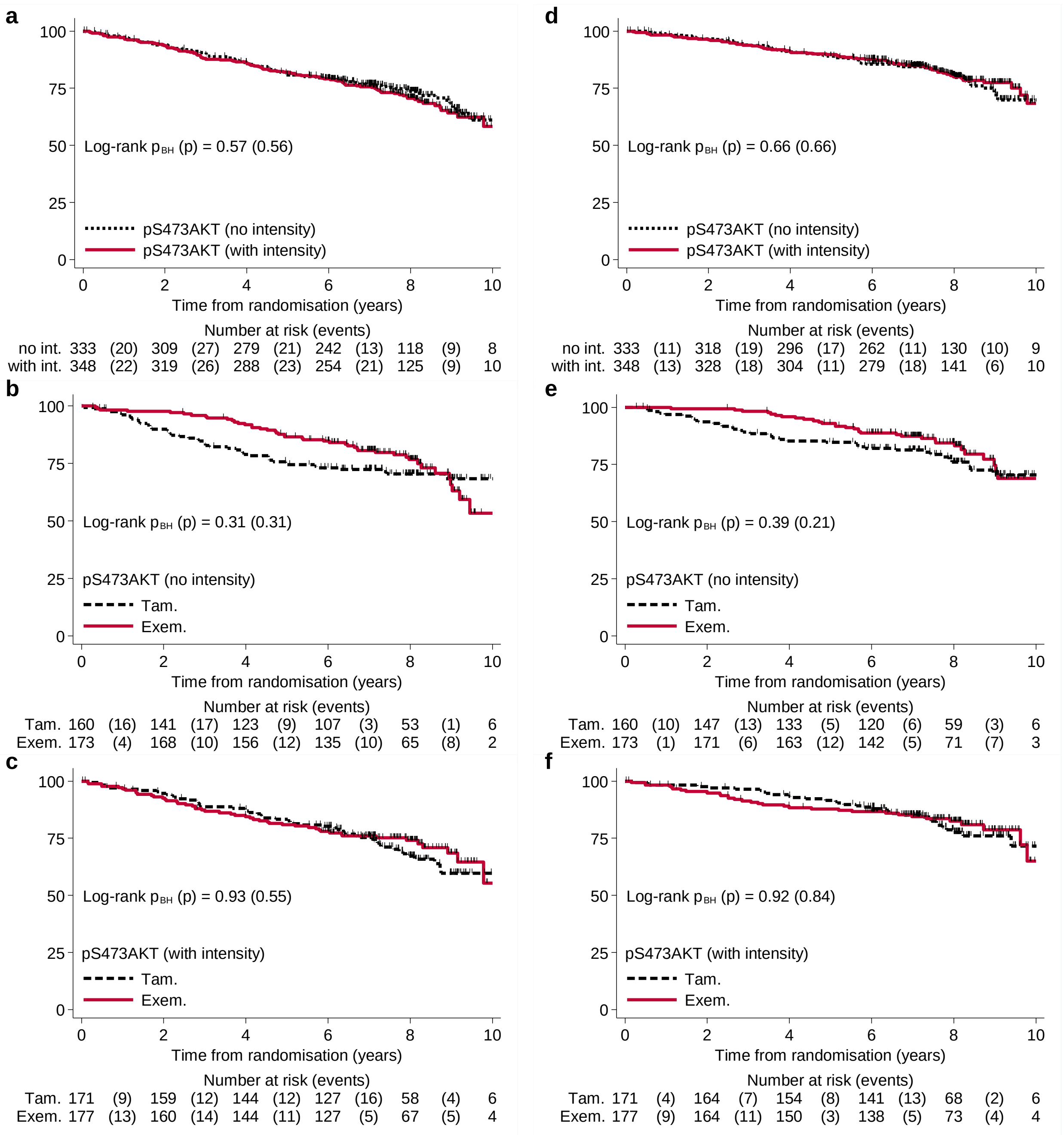

Online Resource 12 Kaplan-Meier estimates for DFS and OS by biological pathways regardless treatment received. **a** DFS and **d** OS estimates by pT202/T204MAPK / pS118ERα groups. **b** DFS and **e** OS estimates by pT308AKT / pS167ERα groups. **c** DFS and **f** OS estimates by pS473AKT / pS167ERα groups. (Abbreviations: p – unadjusted, p_{BH} – Benjamini-Hochberg adjusted)

a

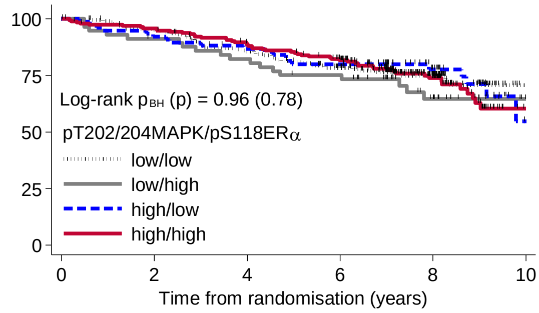

Number at risk (events)

low/low	206	(10)	192	(19)	169	(14)	143	(5)	63	(2)	4
low/high	57	(5)	51	(5)	46	(4)	41	(4)	17	(0)	1
high/low	76	(6)	70	(4)	64	(5)	59	(1)	32	(4)	4
high/high	197	(8)	185	(14)	169	(13)	146	(11)	70	(7)	5

d

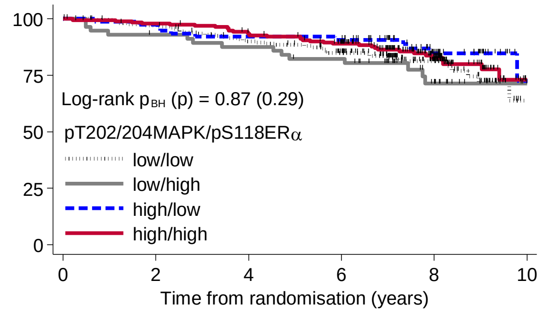

Number at risk (events)

low/low	206	(7)	195	(13)	178	(10)	155	(5)	69	(5)	4
low/high	57	(4)	52	(3)	49	(3)	45	(4)	19	(0)	1
high/low	76	(2)	74	(4)	68	(1)	66	(3)	35	(1)	4
high/high	197	(4)	189	(9)	177	(8)	157	(8)	79	(4)	5

b

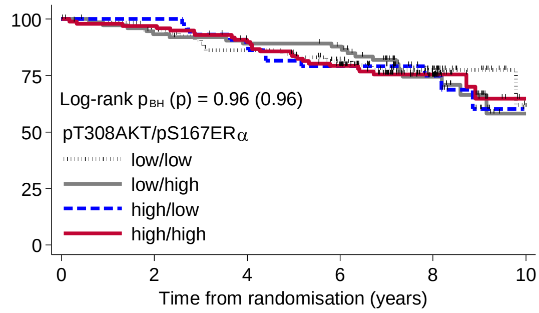

Number at risk (events)

low/low	65	(3)	62	(6)	56	(3)	45	(2)	27	(1)	3
low/high	75	(5)	69	(3)	65	(1)	62	(8)	31	(3)	4
high/low	45	(0)	44	(5)	38	(4)	32	(1)	16	(2)	0
high/high	102	(3)	96	(7)	86	(10)	71	(3)	25	(2)	3

e

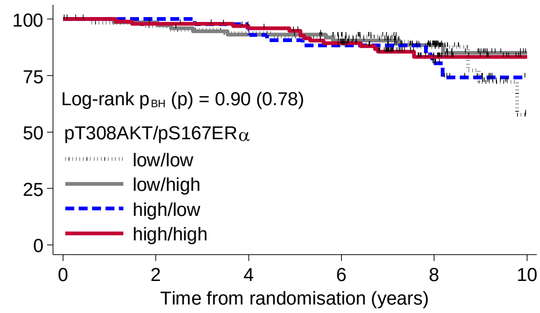

Number at risk (events)

low/low	65	(2)	63	(2)	61	(1)	51	(2)	32	(4)	3
low/high	75	(1)	73	(4)	68	(2)	64	(1)	36	(1)	4
high/low	45	(0)	44	(2)	41	(3)	35	(2)	17	(1)	0
high/high	102	(2)	97	(2)	91	(6)	78	(4)	29	(0)	3

c

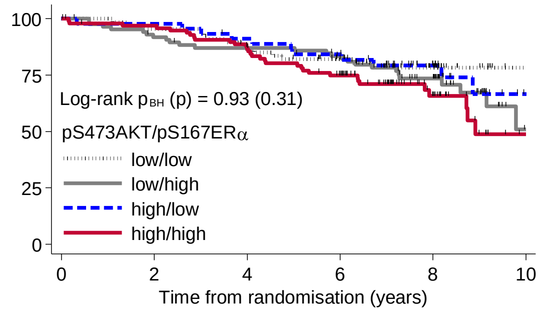

Number at risk (events)

low/low	67	(3)	64	(6)	58	(3)	47	(2)	22	(0)	2
low/high	86	(7)	78	(4)	72	(3)	67	(7)	39	(4)	4
high/low	47	(1)	44	(3)	40	(3)	35	(2)	19	(2)	2
high/high	99	(3)	94	(10)	82	(11)	67	(5)	23	(3)	1

f

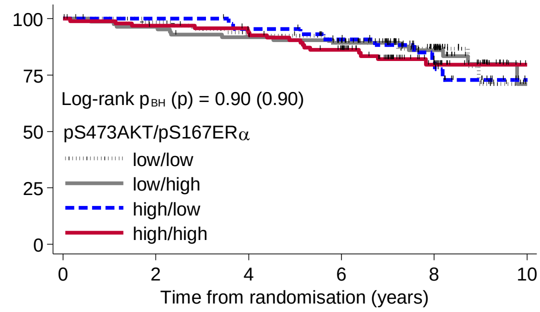

Number at risk (events)

low/low	67	(1)	66	(4)	62	(2)	51	(1)	25	(2)	2
low/high	86	(3)	82	(4)	76	(2)	72	(2)	46	(3)	4
high/low	47	(0)	45	(2)	42	(2)	38	(3)	21	(2)	2
high/high	99	(3)	94	(3)	88	(7)	74	(4)	28	(0)	1

Online Resource 13 Association of biological pathways with disease-free survival (DFS). Univariate Cox PH models for DFS by signalling pathways

	Univariate CoxPH for DFS	
**Biological pathways**	**HR (95 % CI)**[a]	$\mathbf{p_{BH}}$[b]
**pT202/T204MAPK/pS118ERα**		
Low/low	1.00	
Low/high	1.30 (0.76 to 2.23)	0.91
High/low	0.97 (0.57 to 1.63)	0.91
High/high	1.05 (0.71 to 1.55)	0.91
**pT308AKT/pS167ERα**		
Low/low	1.00	
Low/high	1.13 (0.58 to 2.21)	0.91
High/low	1.21 (0.57 to 2.60)	0.91
High/high	1.17 (0.61 to 2.22)	0.91
**pSAKT473/pS167ERα**		
Low/low	1.00	
Low/high	1.30 (0.68 to 2.51)	0.91
High/low	1.98 (0.48 to 2.31)	0.91
High/high	2.71 (0.90 to 3.16)	0.91

[a]CI – confidence interval, [b]p_{BH} - Benjamini-Hochberg adjusted p

Online Resource 14 Association of biological pathways with overall survival (OS). Univariate Cox PH models for OS by signalling pathways

	Univariate CoxPH for OS	
**Biological pathways**	**HR (95 % CI)**[a]	**p_{BH}**[b]
**pT202/T204MAPK/pS118ERα**		
Low/low	1.00	
Low/high	1.24 (0.68 to 2.29)	0.97
High/low	0.63 (0.32 to 1.23)	0.97
High/high	0.80 (0.50 to 1.27)	0.97
**pT308AKT/pS167ERα**		
Low/low	1.00	
Low/high	0.71 (0.29 to 1.71)	0.97
High/low	1.15 (0.46 to 2.87)	0.97
High/high	0.95 (0.43 to 2.09)	0.97
**pSAKT473/pS167ERα**		
Low/low	1.00	
Low/high	0.99 (0.44 to 2.23)	0.97
High/low	1.19 (0.48 to 2.93)	0.97
High/high	1.25 (0.57 to 2.73)	0.97

[a]CI – confidence interval, [b]p_{BH} - Benjamini-Hochberg adjusted p
